# *FADS1* and *FADS2* Gene Polymorphisms Affect Omega-3 and Omega-6 Erythrocyte Fatty Acid Composition and Influence the Association Between Dietary Fatty Acid Intake and Lipid Profile in Brazilian Adults

**DOI:** 10.3390/metabo15120758

**Published:** 2025-11-21

**Authors:** Lais Duarte Batista, Marcelo Macedo Rogero, Flávia Mori Sarti, Marcela Larissa Costa, Jaqueline Lopes Pereira França, João Valentini Neto, Regina Mara Fisberg

**Affiliations:** 1 Department of Nutrition, School of Public Health, University of São Paulo, São Paulo, SP 05403-000, Brazilmmrogero@usp.br (M.M.R.);; 2 School of Arts, Sciences and Humanities, University of São Paulo, São Paulo, SP 03828-000, Brazil; flamori@usp.br; 3 Department of Nutrition, Harvard T.H. Chan School of Public Health, Boston, MA 02115, USA

**Keywords:** erythrocyte fatty acids, *FADS1*, *FADS2*, lipid profile, dietary intake

## Abstract

**Background:** Polymorphisms in the *FADS1* and *FADS2* genes influence fatty acid metabolism. However, evidence of gene–diet interactions in population-based studies from Brazil remains limited. The objective of this study was to examine associations between *FADS1–FADS2* single-nucleotide polymorphisms (SNPs) and erythrocyte fatty acid composition and serum lipid concentrations, as well as to explore potential gene–diet interactions. **Methods**: Data were analyzed from 294 adults (20–93 years) enrolled in the 2015 ISA-Nutrition study. Erythrocyte fatty acid composition and serum lipids were measured using standard enzymatic methods. Dietary intake was assessed by 24 h recalls, and participants were classified into tertiles according to fatty acid intake. Five SNPs were genotyped; *FADS1* rs174546 and *FADS2* rs174570 were prioritized based on linkage disequilibrium. Associations and interactions were assessed using generalized linear models, adjusting for confounders. **Results**: Carriers of the minor alleles for rs174546 and rs174570 had significantly lower erythrocyte levels of long-chain polyunsaturated fatty acids, particularly along the ω-6 pathway, suggesting reduced desaturase activity. The rs174546 TT genotype was associated with higher total, very-low-density lipoprotein cholesterol (VLDL), and non–high-density lipoprotein (non-HDL) cholesterolconcentrations. Higher dietary intakes of docosahexaenoic acid (DHA) or a higher linoleic acid to alpha-linolenic acid ratio(LA/ALA ratio) among these carriers were linked to lower serum lipid levels, indicating gene–diet interactions that attenuate adverse genotype effects. In addition, rs174570 TT carriers showed elevated VLDL concentrations, with a significant dietary interaction observed with the LA/ALA ratio. **Conclusions**: *FADS1* and *FADS2* polymorphisms influence fatty acid metabolism and interact with diet to shape lipid profiles. These findings highlight the importance of considering gene-diet interactions in cardiometabolic risk.

## 1. Introduction

Cardiovascular disease (CVD) remains a leading cause of morbidity and mortality worldwide, with dyslipidemia being one of the primary modifiable risk factors [1]. Dietary fats, particularly polyunsaturated fatty acids (PUFAs), have long been studied for their potential role in modulating lipid metabolism and influencing cardiovascular health [2,3]. Among PUFAs, omega-3 (ω-3) and omega-6 (ω-6) fatty acids have received significant attention due to their distinct biological effects on inflammation, endothelial function, and lipid regulation [4]. Arachidonic acid (AA, ω-6) serves as a precursor to pro-inflammatory eicosanoids, including series-2 prostaglandins, thromboxanes, and leukotrienes. Conversely, eicosapentaenoic acid (EPA, ω-3) primarily contributes to the synthesis of anti-inflammatory mediators such as resolvins and protectins [5].

Epidemiological and interventional studies have suggested that higher intake of long-chain ω-3 PUFAs may be associated with improved lipid profiles and a reduced risk of CVD [6,7]. However, despite numerous investigations into this relationship, the evidence remains inconsistent and at times controversial, highlighting the need for further research to clarify the effects of ω-3 PUFAs on lipid metabolism and cardiovascular outcomes [7]. The most consistent evidence points to a triglyceride-lowering effect of ω-3 supplementation, particularly EPA and docosahexaenoic acid (DHA), a finding supported by both observational studies and randomized controlled trials. However, the effects on other lipid parameters remain less clear and often inconsistent across studies [7]. While some trials have demonstrated cardioprotective benefits of ω-3, others, particularly large-scale recent interventions, have reported null or modest effects, highlighting the debate about their overall efficacy in CVD prevention.

In addition to variability in study design and ω-3 dose or source, inter-individual differences in genetic variation and fatty acid metabolism may help explain these inconsistencies [8,9]. The metabolic conversion of shorter-chain PUFAs, such as alpha-linolenic acid (ALA) and linoleic acid (LA), into their biologically active long-chain derivatives is mainly dependent on the activity of delta-5 and delta-6 desaturase enzymes, encoded by the *FADS1* and *FADS2* genes, respectively [10]. Single-nucleotide polymorphisms (SNPs) within these genes have been shown to modulate the activity of desaturase enzymes involved in PUFA metabolism, thereby influencing the circulating and membrane fatty acid composition [11]. These genetic variants can modulate the effects of dietary intake of ω-3 and ω-6 PUFAs, highlighting a significant gene-diet interaction in lipid metabolism [12]. This interaction may partly explain individual variability in lipid responses and cardiovascular outcomes following PUFA intake [10].

Given the complex interplay between genetic variation, dietary fatty acid intake, and lipid profile, a better understanding of how *FADS1* and *FADS2* polymorphisms affect these relationships is crucial. Therefore, this paper aims to investigate the associations between SNPs in these genes, dietary ω-3 and ω-6 PUFA intake, erythrocyte membrane fatty acid composition, and serum lipid levels, thereby contributing to the emerging field of personalized nutrition and its relevance to cardiovascular risk management.

## 2. Materials and Methods

### 2.1. Study Design and Population

The sample of participants in this study was drawn from the 2015 Health Survey of São Paulo with a focus on Nutrition (2015 ISA-Nutrition), a cross-sectional, population-based study conducted among residents of the urban area of São Paulo, the largest city in Brazil. The primary aim of the study was to investigate the associations between dietary patterns, lifestyle factors, environmental exposures, and biochemical and genetic markers of cardiometabolic diseases. Data collection took place in households throughout 2015 and 2016 [13]. The study initially included 901 individuals with dietary and biochemical measurements, categorized into three age groups: 12–19 years (n = 291), 20–59 years (n = 302), and 60 years and older (n = 308).

**Figure 1:**
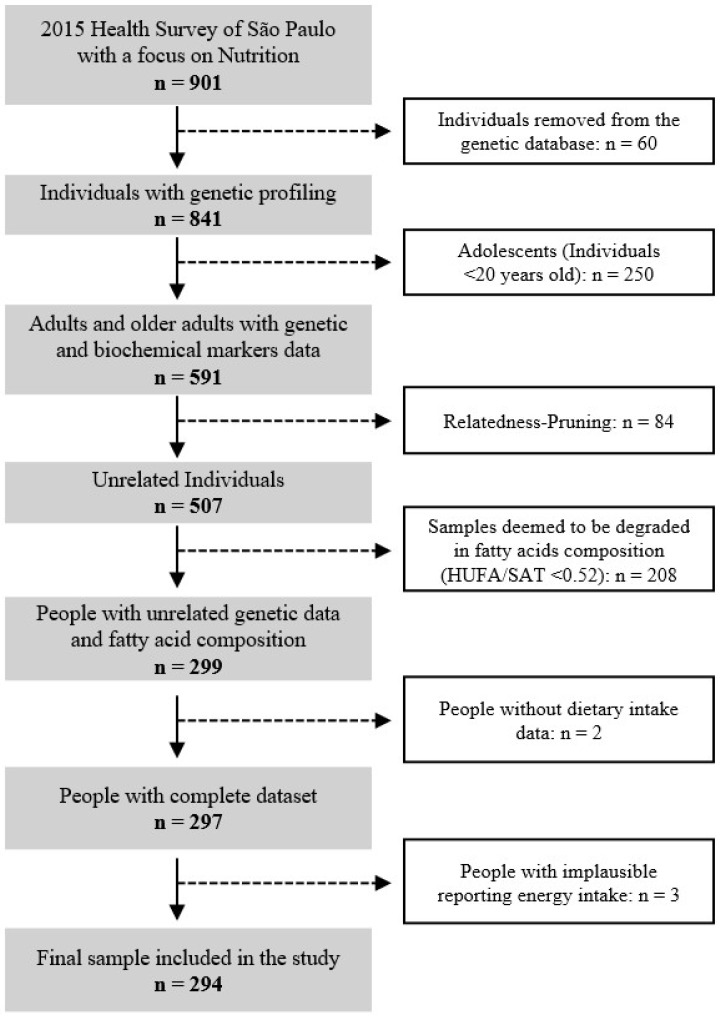
Flowchart of the analytical sample selection from the 2015 ISA-Nutrition survey included in the present study. HUFA/SAT: The ratio of highly unsaturated fatty acids to saturated fatty acids.

For this analysis, we included only adults and older adults with available data on dietary intake, biochemical markers, erythrocyte membrane fatty acid composition, and genotyping. After excluding individuals due to relatedness, sample degradation in fatty acid measurements, and implausible energy intake values, the final analytical sample comprised 294 individuals (Figure 1). The 2015 ISA-Capital study (CAAE nº 36607614.5.0000.5421) and the present study (CAAE nº 51618721.6.0000.5421) were approved by the Research Ethics Committee of the School of Public Health, University of São Paulo. All procedures involving human participants were conducted in accordance with the ethical standards of the Declaration of Helsinki, and written informed consent was obtained from all participants. The Strengthening the Reporting of Observational Studies in Epidemiology (STROBE) checklist for cross-sectional studies was used to guide the writing and reporting of this manuscript [14].

### 2.2. SNP Selection and Genotyping

DNA was quantified from blood samples using the Qubit™ dsDNA BR Assay Kit and the Qubit^®^ 2.0 fluorometer (Thermo Fisher Scientific, Waltham, MA, USA). Genotyping was performed on 864 free-living, healthy individuals using the Axiom™ 2.0 Precision Medicine Research Array (Affymetrix Inc., Santa Clara, CA, USA). Only unrelated individuals were included in the analyses, as determined by the genomic relatedness matrix. Details on genotyping procedures and quality control have been previously published [15]. We selected SNPs within the *FADS1* and *FADS2* genes based on their biological relevance in PUFA metabolism and prior evidence of functional effects or associations with lipid traits. Specifically, the SNPs rs174546 (*FADS1*) and rs174570 (*FADS2*) were chosen because they have been consistently linked to variations in circulating long-chain PUFA levels and lipid profiles in previous genome-wide association studies (GWAS) and candidate gene studies [16,17]. We evaluated Linkage Disequilibrium (LD) patterns of the *FADS1* and *FADS2* loci using data from the 1000 Genomes Project (1KGP) to support SNP selection and interpretation. Given the diverse ancestry of our study population, predominantly composed of individuals with African (AFR), Admixed American (AMR), and European (EUR) backgrounds [15], we specifically considered LD metrics (r^2^) within these reference populations. Genetic ancestry in the population was previously inferred using principal component analysis (PCA) based on genome-wide SNP data. Principal components were computed in PLINK and visualized to assess population structure, allowing the identification of the three main ancestral components in the study population (EUR, AFR, and AMR) by comparison with reference populations from the 1KGP [15]. We evaluated the minor allele frequency (MAF) and tested for Hardy–Weinberg equilibrium (HWE) to assess genotyping quality and population representativeness of the SNPs of interest.

### 2.3. Fatty Acid Composition in Erythrocyte Membranes

The fatty acid composition in erythrocyte membranes was quantified using a gas chromatograph (GC) with a flame ionization detector (Shimadzu, CG-2010, Kyoto, Japan), following a previously published method [18]. Participants fasted for 12 h before blood was collected in EDTA tubes. Red blood cells were separated from plasma by centrifugation (3000× *g*, 4 °C, 10 min) and stored at −80 °C. After cell lysis, the membrane pellet was resuspended in methanol with acetyl chloride to generate fatty acid methyl esters. The mixture was heated, cooled, and then processed to isolate the fatty acids, which were filtered and injected into the GC. Fatty acids were identified by comparing the peaks to an external standard mixture of 37 fatty acids (FAME 37, 47885, Sigma-Aldrich Co, St. Louis, MO, EUA). Each peak was quantified by calculating the area under the peak and expressed as a percentage of the total area under the peaks. A total of 21 fatty acids with clear peak separation under the GC column conditions and that presented meaningful concentrations (>0.1% of total fatty acids) were integrated. To assess sample quality, we calculated the ratio of highly unsaturated fatty acids to saturated fatty acids (HUFA/SAT) [19]. Samples with a ratio below 0.52 were considered degraded and were excluded from the final analysis. To align with the study objectives, we focused only on data for ω-3 and ω-6 fatty acids. Additionally, we calculated the ω-3 index (O3I), defined as the sum of EPA and DHA, expressed as a percentage of total fatty acids [20].

### 2.4. Dietary Intake Assessment

We collected dietary intake data using two 24 h dietary recalls (24HR) on nonconsecutive days, covering different weekdays, weekends, and seasons. Usual intake was estimated using the multiple source method (MSM). The first 24HR was conducted in person at participants’ homes using the Multiple Pass Method [21], while the second was performed by phone following the Automated Multiple Pass Method [22]. Nutritional data were entered into the Nutrition Data System for Research Software (NDSR, version 2021, University of Minnesota) and compared to the Brazilian food composition table [23]. When values differed between databases, they were adjusted to reflect foods consumed by the Brazilian population. Participants with implausible energy intakes were excluded to reduce bias: women reporting less than 500 kcal/day or more than 3500 kcal/day, and men reporting less than 800 kcal/day or more than 4000 kcal/day were removed from the analysis [24]. Fatty acid intake was energy-adjusted using the residual method [24] before inclusion in regression models to control the effect of total energy intake on nutrient consumption. We also used the intake of LA and ALA to calculate the linoleic to alpha-linolenic acid (LA/ALA) ratio, given its known role in erythrocyte membrane composition [25] and lipid markers [26], which are the primary focus of this study.

### 2.5. Biochemical Measurements

Blood samples were collected at the participants’ households by a certified phlebotomist following a 12 h fasting period. The lipid profile was determined by measuring serum concentrations of total cholesterol, low-density lipoprotein (LDL-c), high-density lipoprotein (HDL-c), very-low-density lipoprotein (VLDL), and triglycerides (TG) using enzymatic colorimetric methods with reagents from Cobas—Roche Diagnostics GmbH^®^ (Mannheim, Germany). VLDL levels were estimated by dividing triglyceride concentrations by five. Additionally, non-HDL cholesterol (non-HDL-c) was calculated by subtracting HDL-c from total cholesterol, given its association with cardiovascular disease risk [27].

### 2.6. Other Covariates

We adjusted the models for age, biological sex, use of lipid-lowering medications, body mass index (BMI), and smoking status. In addition to these variables, we also described the population characteristics according to nutritional status, central adiposity, and leisure-time physical activity level (PAL).

Sociodemographic and lifestyle variables were collected through interviewer-administered questionnaires. PAL was assessed using the long version of the International Physical Activity Questionnaire (IPAQ) [28], which is validated for the Brazilian population [29]. Leisure-time physical activity was used to classify participants as meeting or not meeting the World Health Organization (WHO) recommendations for physical activity (i.e., ≥150 min/week vs. <150 min/week) [30].

Body weight, height, and waist circumference were measured in triplicate by trained interviewers using standardized protocols. The average of the three measurements was used to calculate BMI, which was defined as weight (kg) divided by height squared (m^2^). BMI was then categorized according to the WHO cutoff points for adults [31] and the Pan American Health Organization (PAHO) criteria [32] for older adults. Excess weight was defined as having overweight or obesity according to BMI classification, while central adiposity was defined as a waist circumference ≥88 cm for women and ≥102 cm for men [33].

### 2.7. Statistical Analysis

Population characteristics were described using medians and interquartile ranges (IQR) for continuous variables after assessing data distribution with the Shapiro–Wilk test, and absolute and relative frequencies for categorical variables. Differences between age groups were evaluated using the Mann–Whitney test for continuous variables and the Chi-square or Fisher Exact test for categorical variables. To explore differences in ω-3 and ω-6 fatty acid intake by *FADS1* and *FADS2* genotypes, we applied the Kruskal–Wallis test followed by Dunn’s post hoc test for pairwise multiple comparisons. We then examined the distribution of erythrocyte ω-3 and ω-6 fatty acid composition across their dietary intake tertiles, stratified by genotype. Generalized linear models (GLMs) were used to assess the associations between dietary intake of fatty acids and lipid profile markers. Models were adjusted for potential confounders, including age, biological sex, BMI, smoking status, and use of lipid-lowering medications. Additionally, the models were adjusted for population ancestry, based on the three reference populations from the 1KGP (AFR, AMR, and EUR), which were identified by Pereira et al. [15] as representative of the ancestry composition of this study population. In addition to main effects, SNP genotypes were included as independent variables, and interaction terms between dietary fatty acid intake and SNPs were examined to assess potential gene–diet interactions influencing lipid outcomes. All statistical analyses were performed in RStudio (version 2024.12.0), with a significance level set at 5%.

## 3. Results

Participants were predominantly male (57.5%), with a mean (SD) age of 58.4 (15.0) years, and the majority were older adults (≥60 years; 55.4%). Most characteristics did not significantly differ between age groups (Table 1). However, older adults reported a lower usual energy intake (*p* = 0.003), accompanied by reduced intake of total ω-3 fatty acids (*p* = 0.046), ALA (*p* = 0.036), and linoleic acid (*p* = 0.016). The LA/ALA ratio was also slightly lower in this group (*p* = 0.016). Age-related differences were observed in nutritional status (*p* = 0.001), central adiposity (*p* = 0.005), and medication use (*p* < 0.001). Older people had a lower prevalence of excess body weight but a higher prevalence of central obesity and statins/lipid-lowering medication use.

The genotype distributions, MAF, and HWE *p*-values for the five *FADS1-FADS2* SNPs are presented in Table 2. All genotype frequencies were consistent with the HWE (*p* > 0.05), suggesting no significant deviations from expected proportions under equilibrium conditions. MAFs ranged from 20.1% to 38.9%, reflecting adequate genetic variability within the study population. Given the high linkage disequilibrium observed among the three *FADS1* SNPs (Supplementary Figure S1) in the 1KGP, only one representative variant (rs174546) was selected for further association analyses. This SNP was selected based on the availability of complete genotype data for all 294 participants. In contrast, rs174570 showed weaker correlations with the other *FADS1* and *FADS2* variants and was therefore retained to capture potentially distinct genetic effects. rs174583 was excluded due to its strong correlation with the other SNPs, which would have introduced redundancy without providing additional insight. This selection approach helped to minimize redundancy due to linkage disequilibrium and preserved statistical power for phenotype-based analyses.

We observed significant differences in erythrocyte membrane fatty acid composition across genotypes for both the FADS1 (rs174546) and FADS2 (rs174570) genes (Table 3). For rs174546, carriers of the TT genotype had significantly lower levels of docosapentaenoic acid (DPA, C22:5 n-3) compared to those with the CC and CT genotypes (*p* = 0.010). In contrast, no significant differences were observed for ALA, EPA, DHA, or the overall O3I. Genotype-related differences were more pronounced for ω-6 fatty acids. Significant variation was observed in LA, dihomo-γ-linolenic acid (DGLA), arachidonic acid (AA), and docosatetraenoic acid across rs174546 genotypes (*p* < 0.01). TT carriers showed lower levels of AA and higher levels of LA and DGLA, suggesting reduced conversion efficiency along the ω-6 pathway.

Similar trends were observed for rs174570 (*FADS2*), where individuals with the TT genotype had significantly higher concentrations of DGLA (*p* < 0.001) and LA (*p* = 0.001), and lower levels of AA (*p* < 0.001) compared to the CC and CT genotypes. These findings are consistent with impaired desaturase activity in minor allele carriers and support the role of *FADS* polymorphisms in modulating long-chain PUFA biosynthesis and metabolism, particularly within the ω-6 pathway.

**Figure 2:**
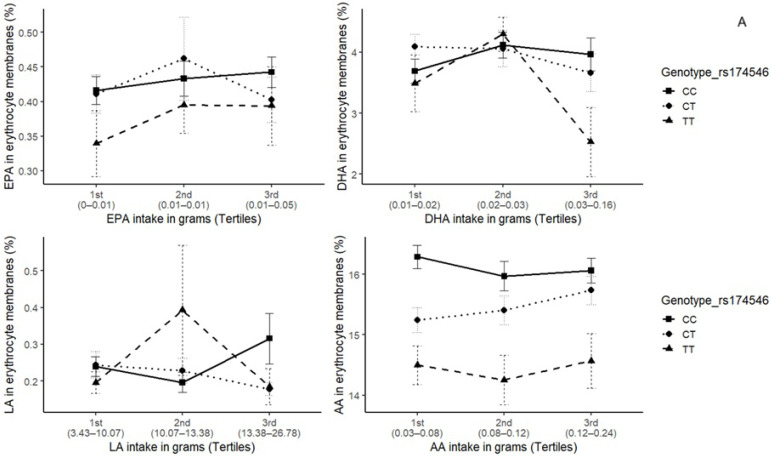

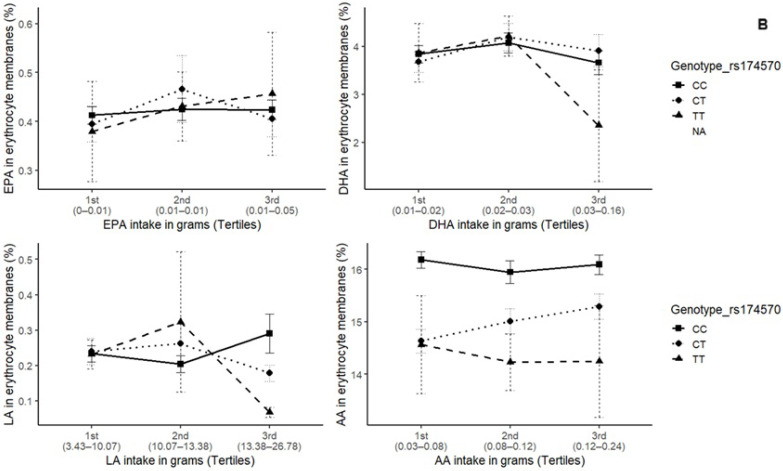
ω-3 and ω-6 fatty acid composition in erythrocyte membranes by dietary intake tertiles and *FADS1/FADS2* polymorphism genotypes. EPA: Eicosapentaenoic; DHA: Docosahexaenoic; LA: Linoleic; AA: Arachidonic. Panels (**A**): *FADS1* rs174546 and (**B**) *FADS2* rs174570 polymorphisms. Error bars indicate the standard error of the mean.

Further investigation of the potential gene-diet interactions influencing erythrocyte fatty acid composition was conducted by stratifying participants according to dietary intake tertiles and examining fatty acid profiles according to *FADS1* rs174546 and *FADS2* rs174570 genotypes (Figure 2). This approach enabled us to investigate whether individuals with different genotypes exhibited distinct metabolic responses to varying levels of dietary intake. For both SNPs, carriers of the minor allele (CT and TT genotypes) exhibited lower EPA and DHA levels across most intake tertiles, particularly at higher ω-3 intakes, suggesting reduced desaturase activity and less efficient conversion of precursors into long-chain PUFA. For ω-6 fatty acids, AA levels were consistently lower in TT carriers, regardless of AA intake, reinforcing the hypothesis that *FADS1/FADS2* SNPs modulate the efficiency of long-chain PUFA biosynthesis from dietary precursors, with impaired desaturase activity in minor allele carriers.

We examined whether the associations between dietary ω-3 and ω-6 fatty acids intake and lipid profile differed according to *FADS1* (rs174546) and *FADS2* (rs174570) genotypes to further explore gene-diet interactions. Models showing statistically significant interactions or main effects are presented in Table 4. Notably, we observed a significant interaction between DHA intake and the rs174546 TT genotype for total (*p* = 0.031), VLDL (*p* = 0.032), and non-HDL (*p* = 0.016) cholesterol levels, suggesting that carriers of the minor allele may exhibit an attenuated lipid response to higher DHA intake.

Regarding rs174570, we observed fewer significant interactions, but some findings suggest a potential modulatory role in lipid metabolism. Individuals with the TT genotype exhibited significantly higher VLDL concentrations (*p* = 0.020), indicating a possible impact of this variant on VLDL metabolism. Additionally, a significant interaction between the TT genotype and the LA/ALA ratio was observed for VLDL (*p* = 0.045), suggesting that minor allele carriers may exhibit differential responses to the dietary ω-6 to ω-3 balance. The results indicate a modest role of rs174570 in influencing lipid profiles, particularly VLDL cholesterol, in response to dietary fatty acid composition. These findings support a modulatory role for both *FADS1* and *FADS2* polymorphisms in lipid metabolism, suggesting that individuals with different genotypes may respond differently to specific PUFA dietary intake.

## 4. Discussion

In this study, we investigated the influence of *FADS1* and *FADS2* polymorphisms on erythrocyte fatty acid composition and serum lipid profiles in a Brazilian population, with a focus on gene–diet interactions. Our results suggest that the minor alleles of rs174546 and rs174570 SNPs are associated with lower levels of erythrocyte membrane PUFAs, particularly within the ω-6 pathway, potentially reflecting reduced activity of the desaturase enzyme. The rs174546 TT genotype was associated with higher levels of lipid markers; however, higher dietary intake of LA/ALA or DHA appeared to attenuate these effects, indicating a possible gene–diet interaction influencing lipid metabolism. In contrast, the rs174570 TT genotype was also linked to elevated serum lipid markers and with a significant interaction with LA/ALA dietary intake in VLDL. Biologically, *FADS1* and *FADS2* encode the delta-5 and delta-6 desaturase enzymes, respectively, which are essential for the biosynthesis of long-chain PUFAs [34]. Impaired activity of these enzymes, due to genetic variation, can disrupt PUFA metabolism and lipid homeostasis, thereby influencing cardiovascular risk factors. Our findings underscore the importance of considering both genetic variation and dietary intake when assessing lipid metabolism and its associated health outcomes.

Variants in *FADS1*, such as rs174546, have been associated with enzymatic efficiency in converting essential fatty acids into their long-chain derivatives, potentially leading to altered serum lipid patterns [35]. Our findings extend this knowledge by providing evidence from a Latin American population, an underrepresented group in genetic epidemiology studies. The gene–diet interaction observed for rs174546 suggests that higher dietary intake of PUFAs can mitigate the adverse lipid effects associated with this genotype. In contrast, the fewer significant interactions for rs174570 may indicate locus-specific differences in response to dietary fatty acids [12,36]. Both variants are located in non-coding regions of the gene and are therefore unlikely to alter the enzyme’s structure. Instead, they may influence regulatory processes, such as transcriptional activity, mRNA splicing or stability, and post-transcriptional control, which could explain their distinct functional effects. This supports the concept that personalized nutrition strategies tailored to genetic background may help optimize cardiometabolic health.

Most participants were older adults who reported lower energy and ω-3 fatty acid intakes compared to younger individuals. This aligns with known changes in dietary habits and nutrient requirements that occur with aging. The reduced intake of ω-3, ALA, and linoleic acid may result from factors such as altered appetite [37], food preferences, or limited access to nutrient-rich foods [38], which could impact fatty acid status and cardiometabolic health in this population. Additionally, older adults are more likely to underreport energy intake [39], which may partly explain the observed lower energy and nutrient intake values. Although findings across studies are inconsistent, the lower LA/ALA ratio observed in older adults may also contribute to alterations in fatty acid metabolism [25,40], as this balance affects downstream synthesis of long-chain PUFAs, potentially modifying inflammatory and lipid pathways [41]. Despite a lower prevalence of excess body weight, older adults showed a higher prevalence of central adiposity and greater use of statins and other lipid-lowering medications. This pattern aligns with epidemiological evidence indicating that aging is associated with a shift in body fat distribution toward visceral fat accumulation [42], which is more strongly linked to cardiovascular risk than overall adiposity [43]. The increased use of lipid-lowering medications likely reflects the higher burden of dyslipidemia and cardiovascular disease in this group [44]. Together, these findings underscore the importance of considering age-related differences in diet, body composition, and medication when interpreting gene–diet interactions and lipid profiles. Adjusting for these factors is essential to minimize confounding and to elucidate better the mechanisms linking *FADS* polymorphisms, dietary intake, and lipid metabolism across the lifespan.

The genotype-related differences in erythrocyte fatty acid composition support the biological role of *FADS1* and *FADS2* polymorphisms in regulating long-chain PUFA biosynthesis [34]. The consistently lower levels of downstream metabolites such as AA (ω-6), along with EPA and DPA (ω-3), combined with higher levels of LA among TT carriers of both rs174546 and rs174570, are consistent with reduced desaturase enzyme activity in individuals with minor alleles [45,46]. Notably, these genotype-related differences persisted across varying levels of dietary PUFA intake, suggesting that genetic variation limits endogenous conversion efficiency regardless of precursor availability. This has important implications for personalized nutrition strategies, as individuals with impaired desaturase activity may benefit more from direct dietary sources of preformed long-chain PUFAs (e.g., from fatty fish or EPA/DHA supplements) rather than from plant-derived precursors, such as ALA. The consistent findings across both SNPs strengthen the evidence for functional effects of *FADS* variants and their relevance for lipid metabolism and cardiometabolic health. Additionally, the genotype–phenotype pattern observed for both *FADS1* and *FADS2* polymorphisms suggests an additive or semi-dominant effect rather than a strictly recessive model. Heterozygous carriers (CT) exhibited intermediate levels of erythrocyte PUFA composition compared with the CC and TT genotypes, indicating a graded influence of the T allele on fatty acid desaturation. This pattern is consistent with previous reports describing additive effects of *FADS* region variants on PUFA biosynthesis and reinforces the notion that even partial reductions in desaturase activity may have nutritional and clinical relevance [16].

The strengths of our study include rigorous quality control of high-quality genotyping data, with all SNPs in HWE and sufficient allele frequency variation. Selecting representative SNPs based on linkage disequilibrium patterns and complete data minimized redundancy and preserved statistical power. Importantly, fatty acid composition was measured in erythrocyte membranes, reflecting the long-term fatty acid status of red blood cells over their lifespan and providing a more stable biomarker than plasma measurements [20]. We applied strict methods to detect and exclude degraded samples [19], ensuring high sample quality despite the modest sample size. Additionally, the population-based sample from an underrepresented Latin American group contributes valuable insights to genetic epidemiology. However, limitations of the study include the cross-sectional design, which restricts causal inference. In addition, the dietary assessment via 24 h recalls may not adequately capture sporadically consumed foods, such as ω-3-rich items [47]. Using a food frequency questionnaire (FFQ) could improve estimates of habitual intake. Furthermore, the modest sample size and focus on two SNPs may limit generalizability and the ability to detect some gene–diet interactions. The small number of individuals carrying the TT genotype of rs174570 (n = 15) may limit the statistical power to detect gene–diet interactions and should therefore be considered a study limitation. However, both the HWE and MAF were consistent with expectations and comparable to those reported in similar populations, supporting the reliability of the genetic data. Additionally, information on genetic variants in ELOVL2 and ELOVL5 was not available in the dataset; therefore, potential effects of elongase gene variation on long-chain PUFA levels could not be evaluated.

Future research should consider employing longitudinal designs to establish more robust causal relationships between *FADS* variants, diet, and lipid metabolism. Larger, more diverse cohorts would improve the power to detect gene–diet interactions and explore additional genetic variants. Incorporating more precise dietary assessment methods and biomarker measurements could reduce bias and better quantify PUFA intake and status. Ultimately, intervention studies examining personalized dietary recommendations based on *FADS* genotypes could provide direct evidence for the clinical utility of genotype-guided nutrition strategies in improving cardiometabolic health.

## 5. Conclusions

In conclusion, our findings highlight the modulatory role of *FADS1* and *FADS2* genetic variants in long-chain PUFA biosynthesis and demonstrate genotype-dependent differences in lipid responses to dietary PUFA intake. This gene–diet interaction may underlie individual variability in fatty acid status and metabolic risk, emphasizing the importance of considering genetic background in nutritional strategies. However, genetics represents only one of multiple determinants of metabolic health. Behavioral and environmental factors, such as overall diet quality, physical activity, and lifestyle habits, also play key and complementary roles. Together, these findings contribute to the growing evidence of a nutrigenetic component in lipid metabolism, which could inform more comprehensive and personalized dietary recommendations aimed at improving cardiometabolic health.

## Figures and Tables

**Table 1:** Lipid profile, dietary, and sociodemographic characteristics stratified by age groups.

	Total Population (n = 294)	Age Groups		
		20–59 Years (n = 131)	60+ Years (n = 163)	
	Median (IQR)	Median (IQR)	Median (IQR)	*p*
Total cholesterol (mg/dL)	187 (57)	189 (52)	186 (59)	0.301
HDL cholesterol (mg/dL)	43 (19)	41.5 (20)	44 (17)	0.315
LDL cholesterol (mg/dL)	113 (49)	118.5 (44)	112 (53)	0.320
VLDL cholesterol (mg/dL)	24 (15)	24 (16)	24 (14)	0.952
Triglycerides (mg/dL)	120 (73.5)	121 (77)	118 (72)	0.902
non-HDL cholesterol (mg/dL)	144 (60)	148.5 (56)	138 (60)	0.177
Adjusted usual energy intake (kcal/day)	1679 (595)	1772 (621)	1615 (534)	**0.003**
Dietary total fat intake (%)	30.9 (6.7)	31.4 (7.0)	30.7 (6.7)	0.112
Total polyunsaturated fatty acids intake (%)	7.3 (2.1)	7.2 (2.1)	7.4 (2.1)	0.717
Total ω-3 fatty acids usual intake (g)	1.6 (0.7)	1.7 (0.7)	1.5 (0.7)	**0.046**
Eicosapentaenoic (EPA) usual intake (g)	0.01 (0.01)	0.01 (0.01)	0.01 (0.01)	0.836
Docosahexaenoic (DHA) usual intake (g)	0.03 (0.01)	0.03 (0.02)	0.03 (0.01)	0.291
Alpha-Linolenic acid (ALA) usual intake (g)	1.5 (0.6)	1.7 (0.6)	1.5 (0.5)	**0.036**
Linoleic acid usual intake (g)	12.0 (5.3)	12.6 (5.7)	11.5 (4.6)	**0.016**
Linoleic/ALA ratio	7.7 (1.1)	7.8 (1.1)	7.6 (1.1)	**0.016**
**Sex—n (%)** *				
Male	169 (57.5)	75 (57.2)	94 (57.7)	0.943
Female	125 (42.5)	56 (42.8)	69 (42.3)	
**Nutritional Status—n (%)** *				
Without overweight/obesity	135 (46.1)	46 (35.1)	89 (54.9)	**0.001**
Excess weight (overweight or obesity)	158 (53.9)	85 (64.9)	73 (45.1)	
**Central adiposity—n (%)** *				
<88 cm (women) or <102 cm (men)	132 (45.5)	70 (54.7)	62 (38.3)	**0.005**
≥88 cm (women) or ≥102 cm (men)	158 (54.5)	58 (45.3)	100 (61.7)	
**Leisure-Time Physical Activity—n (%)** *				
<150 min/week	241 (83.1)	109 (83.9)	132 (82.5)	0.761
≥150 min/week	49 (16.9)	21 (16.1)	28 (17.5)	
**Smoking status—n (%)** *				
Never	170 (58.0)	80 (61.1)	90 (55.6)	0.448
Current or past	123 (42.0)	51 (38.9)	72 (44.4)	
**Statins or antilipidemic use—n (%)** **				
No	263 (89.5)	127 (96.9)	136 (83.4)	**<0.001**
Yes	31 (10.5)	4 (3.1)	27 (16.6)	

IQR: Interquartile range. HDL: High-density lipoprotein. LDL: Low-density lipoprotein. VLDL: Very low-density lipoprotein. *p*: Mann–Whitney U test for comparisons between age groups. * Chi-square or ** Fisher’s exact test for association with age groups.

**Table 2:** Characteristics of *FADS1* and *FADS2* single-nucleotide polymorphisms in a sample of Brazilians.

SNP	Gene	M/m Alleles	Genotype			MAF (%)	HWE
			CC	CT	TT		
rs174537	*FADS1*	C/T	151 (51.5%)	109 (37.2%)	33 (11.3%)	29.9%	0.069
rs174546	*FADS1*	C/T	151 (51.4%)	110 (37.4%)	33 (11.2%)	29.9%	0.070
rs174556	*FADS1*	C/T	161 (54.8%)	109 (37.1%)	24 (8.2%)	26.7%	0.372
rs174570	*FADS2*	C/T	190 (64.9%)	88 (30.0%)	15 (5.1%)	20.1%	0.275
rs174583	*FADS2*	C/T	117 (39.8%)	125 (42.5%)	52 (17.7%)	38.9%	0.067

SNP: Single-nucleotide polymorphisms; M/m: Major and minor alleles; MAF: Minor allele frequency. HWE: Hardy–Weinberg equilibrium exact test.

**Table 3:** Fatty acid percentage composition in erythrocyte membranes in Brazilian adults stratified by *FADS1* and *FADS2* gene polymorphisms.

	rs174546—*FADS1*				rs174570—*FADS2*			
ω-3 Fatty Acids	CC (n = 151)	CT (n = 110)	TT (n = 33)	*p*	CC (n = 190)	CT (n = 88)	**TT (n = 15)**	***p***
Alpha-Linolenic (C18:3 ω3)	0.15 (0.10)	0.16 (0.13)	0.13 (0.10)	0.285	0.15 (0.10)	0.15 (0.14)	0.18 (0.15)	0.854
Eicosapentaenoic (C20:5 ω3)	0.41 (0.16)	0.40 (0.16)	0.35 (0.23)	0.057	0.40 (0.16)	0.36 (0.14)	0.37 (0.39)	0.199
Docosapentaenoic (C22:5 ω3)	2.24 (0.51)	2.30 (0.47)	2.07 (0.40)	**0.010 b,c**	2.27 (0.48)	2.19 (0.51)	2.02 (0.35)	0.081
Docosahexaenoic (C22:6 ω3)	3.83 (1.58)	3.93 (1.59)	3.80 (1.34)	0.636	3.87 (1.59)	3.86 (1.52)	3.72 (1.50)	0.758
ω-3 Index (EPA + DHA)	4.29 (1.74)	4.25 (1.59)	4.09 (1.67)	0.645	4.28 (1.74)	4.21 (1.54)	4.03 (1.75)	0.811
**ω-6 Fatty acids**								
Linoleic (C18:2 ω6)	9.42 (1.93)	9.95 (2.00)	10.3 (1.67)	**0.006 a,b**	9.40 (1.94)	10.2 (1.77)	10.7 (1.96)	**0.001 a**
Gamma-Linolenic (C18:3 ω6)	0.18 (0.15)	0.19 (0.18)	0.16 (0.17)	0.715	0.18 (0.16)	0.19 (0.16)	0.17 (0.22)	0.604
Eicosadienoic (C20:2 ω6)	0.23 (0.15)	0.25 (0.16)	0.26 (0.09)	0.300	0.23 (0.16)	0.26 (0.14)	0.26 (0.20)	0.208
Dihomo-y-linolenic (C20:3 ω6)	1.57 (0.44)	1.84 (0.44)	2.24 (0.45)	**<0.001 a,b,c**	1.64 (0.45)	1.89 (0.57)	2.15 (0.54)	**<0.001 a,b**
Arachidonic (C20:4 ω6)	16.0 (1.88)	15.3 (1.89)	14.7 (1.83)	**<0.001 a,b,c**	16.0 (1.83)	14.9 (1.51)	14.6 (2.38)	**<0.001 a,b**
Docosatetraenoic (C22:4 ω6)	3.58 (0.77)	3.41 (0.65)	3.21 (0.87)	**0.004 b**	3.53 (0.74)	3.40 (0.59)	3.34 (1.26)	0.075
**Total PUFA %**	37.9 (3.05)	37.9 (2.78)	36.9 (2.63)	0.201	37.9 (2.90)	37.9 (2.96)	36.8 (1.40)	0.220

Values are presented as median (interquartile range—IQR). PUFA: Polyunsaturated fatty acids. EPA: Eicosapentaenoic. DHA: Docosahexaenoic. *p*: Kruskal–Wallis with Dunn post hoc test for pairwise multiple comparisons. a: CC vs. CT; b: CC vs. TT; c: CT vs. TT.

**Table 4:** Genotype-diet interactions of *FADS1* and *FADS2* polymorphisms on serum lipid concentrations.

	FADS1 (rs174546)			FADS2 (rs174570)		
Total Cholesterol (mg/dL)	β	SE	*P*	β	SE	*P*
*LA/ALA ratio*	−0.01	0.02	0.534	−0.01	0.01	0.642
Genotype—CT	0.31	0.19	0.103	0.49	0.18	**0.008**
Genotype—TT	0.55	0.27	**0.041**	0.57	0.33	0.083
Interaction CT vs. LA/ALA ratio	−0.04	0.02	0.092	−0.06	0.02	**0.007**
Interaction TT vs. LA/ALA ratio	−0.07	0.03	**0.031**	−0.07	0.04	0.083
* DHA_Adjusted for energy*	−0.43	1.07	0.689	-		
Genotype—CT	0.06	0.07	0.397			
Genotype—TT	0.17	0.10	0.081			
Interaction CT vs. DHA intake	−2.08	1.99	0.299			
Interaction TT vs. DHA intake	−6.11	2.83	**0.031**			
**VLDL (mg/dL)**						
*LA/ALA ratio*	-			−0.04	0.04	0.273
Genotype—CT				0.27	0.52	0.603
Genotype—TT				2.01	0.86	**0.020**
Interaction CT vs. LA/ALA ratio				−0.01	0.07	0.937
Interaction TT vs. LA/ALA ratio				−0.22	0.11	**0.045**
*DHA_Adjusted for energy*	−4.64	2.79	0.097	−5.80	2.5	**0.022**
Genotype—CT	0.03	0.17	0.857	0.13	0.17	0.466
Genotype—TT	0.85	0.26	**0.001**	0.73	0.31	**0.021**
Interaction CT vs. DHA intake	3.52	4.91	0.473	3.65	4.99	0.464
Interaction TT vs. DHA intake	−15.6	7.30	**0.032**	−11.7	8.83	0.186
**LDL cholesterol (mg/dL)**						
LA/ALA ratio	−0.01	0.02	0.868	−0.01	0.02	0.908
Genotype—CT	0.62	0.27	**0.021**	0.82	0.26	**0.002**
Genotype—TT	0.45	0.39	0.245	0.23	0.47	0.635
Interaction CT vs. LA/ALA ratio	−0.08	0.03	**0.019**	−0.11	0.03	**0.001**
Interaction TT vs. LA/ALA ratio	−0.07	0.05	0.173	−0.03	0.06	0.594
**Non-HDL cholesterol (mg/dL)**						
*LA/ALA ratio*	−0.01	0.02	0.481	−0.01	0.02	0.604
Genotype—CT	0.49	0.26	0.064	0.74	0.25	**0.004**
Genotype—TT	0.72	0.37	**0.049**	0.72	0.45	0.106
Interaction CT vs. LA/ALA ratio	−0.06	0.03	0.074	−0.09	0.03	**0.005**
Interaction TT vs. LA/ALA ratio	−0.09	0.05	0.058	−0.09	0.06	0.129
*DHA_Adjusted for energy*	−1.05	1.45	0.468	-		
Genotype—CT	0.07	0.09	0.454			
Genotype—TT	0.32	0.13	**0.016**			
Interaction CT vs. DHA intake	−1.44	2.68	0.589			
Interaction TT vs. DHA intake	−9.24	3.82	**0.016**			

Generalized Linear Models with family Gamma and link log. All models were adjusted for age, sex, BMI, lipid-lowering medication, population ancestry, and smoking. VLDL: Very low-density lipoprotein. Non-HDL: Total cholesterol minus high-density lipoprotein cholesterol. LDL: Low-density lipoprotein. LA/ALA: Linoleic to alpha-linolenic acid ratio. β: Regression coefficient. SE: Standard Error.

## Data Availability

The data presented in this study are available on request from the corresponding author. The data are not publicly available due to privacy and ethical restrictions.

## Supplementary Materials

The following supporting information can be downloaded at: https://www.mdpi.com/article/10.3390/metabo15120758/s1, Supplementary Figure S1: Linkage Disequilibrium (LD) heatmap across populations from different genetic ancestries in the 1000 Genomes Project.

## Abbreviations

The following abbreviations are used in this manuscript:

24HR	24-Hour Dietary Recall
AA	Arachidonic Acid
AFR	African
ALA	Alpha-Linolenic Acid
AMR	Admixed American
BMI	Body Mass Index
CVD	Cardiovascular Disease
DHA	Docosahexaenoic Acid
EPA	Eicosapentaenoic Acid
EUR	European
*FADS1/FADS2*	Fatty Acid Desaturase 1/Fatty Acid Desaturase 2
FFQ	Food Frequency Questionnaire
GC	Gas Chromatograph
GLM	Generalized Linear Model
GWAS	Genome-Wide Association Study
HDL-c	High-Density Lipoprotein Cholesterol
HUFA/SAT	Highly Unsaturated Fatty Acids-to-Saturated Fatty Acids Ratio
HWE	Hardy–Weinberg Equilibrium
IPAQ	International Physical Activity Questionnaire
IQR	Interquartile Range
ISA-Nutrition	Health Survey of São Paulo with a focus on Nutrition
LA	Linoleic Acid
LD	Linkage Disequilibrium
LDL-c	Low-Density Lipoprotein Cholesterol
MAF	Minor Allele Frequency
MSM	Multiple Source Method
NDSR	Nutrition Data System for Research
Non-HDL-c	Non-High-Density Lipoprotein Cholesterol
O3I	Omega-3 Index
PAHO	Pan American Health Organization
PAL	Physical Activity Level
PUFA	Polyunsaturated Fatty Acid
SNP	Single-Nucleotide Polymorphism
STROBE	Strengthening the Reporting of Observational Studies in Epidemiology
TG	Triglycerides
VLDL	Very-Low-Density Lipoprotein
WHO	World Health Organization
ω-3	Omega-3 Fatty Acid
ω-6	Omega-6 Fatty Acid
