# Supplementary material for: FADS1 and FADS2 Gene Polymorphisms Affect Omega-3 and Omega-6 Erythrocyte Fatty Acid Composition and Influence the Association Between Dietary Fatty Acid Intake and Lipid Profile in Brazilian Adults
Source: Metabolites. 2025 Nov 21;15(12):758. doi: 10.3390/metabo15120758 (PMC12735183; doi:10.3390/metabo15120758)
Supplement: Supplementary file 1 [file metabolites-15-00758-s001.zip › metabolites-3961760-supplementary.pdf]

FADS1 and *FADS2* Gene Polymorphisms Affect Omega-3 and Omega-6 Erythrocyte Fatty Acid Composition and Influence the Association Between Dietary Fatty Acid Intake and Lipid Profile in Brazilian Adults

Lais Duarte Batista [1], Marcelo Macedo Rogero [1], Flávia Mori Sarti [2], Marcela Larissa Costa [1,3], Jaqueline Lopes Pereira França [1], João Valentini Neto [1,3] and Regina Mara Fisberg [1]

1 Department of Nutrition, School of Public Health, University of São Paulo, São Paulo, SP 05403-000, Brazil

2 School of Arts, Sciences and Humanities, University of São Paulo, São Paulo, SP 03828-000, Brazil

3 Department of Nutrition, Harvard T.H. Chan School of Public Health, Boston, MA 02115, USA

Supplementary Figure S1. Linkage Disequilibrium (LD) heatmap across populations from different genetic ancestries in the 1000 Genomes Project.

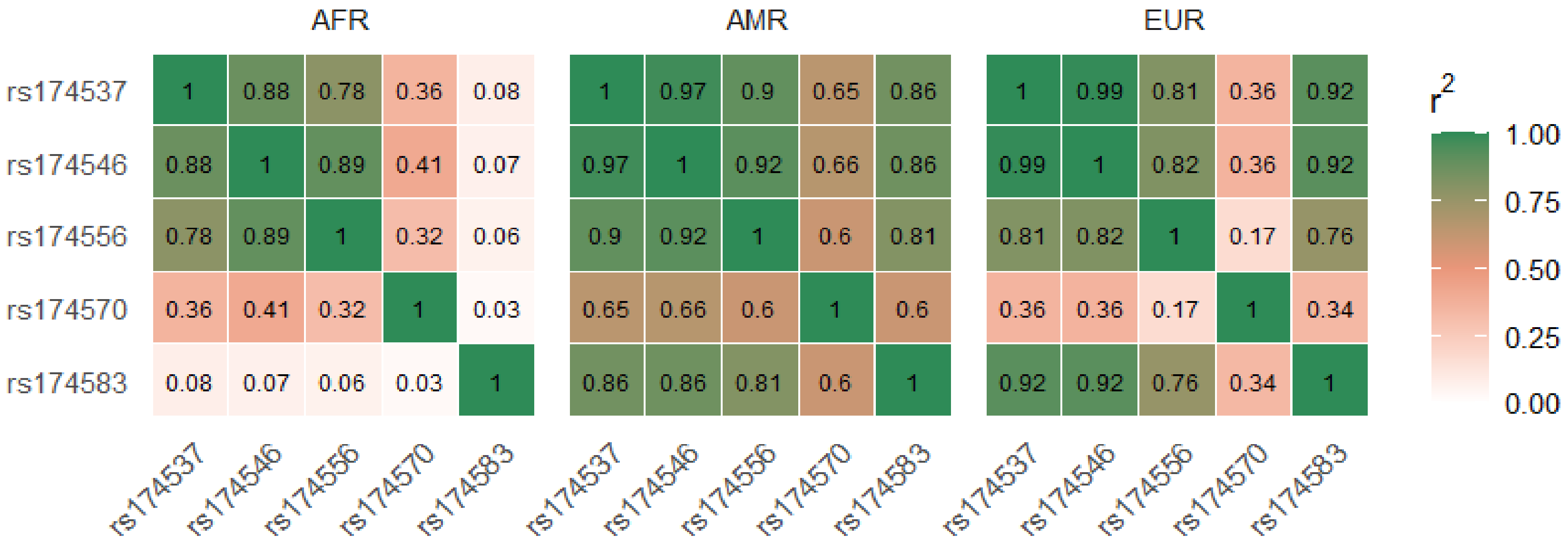

EUR - Europeans; AMR - Admixed Americans; AFR – Africans.
